# Malignancies After Lung Transplantation

**DOI:** 10.3389/ti.2024.12127

**Published:** 2024-09-09

**Authors:** Caroline Stenman, Andreas Wallinder, Erik Holmberg, Kristjan Karason, Jesper Magnusson, Göran Dellgren

**Affiliations:** ^1^ Transplant Institute, Sahlgrenska University Hospital, Gothenburg, Sweden; ^2^ Department Cardiothoracic Surgery, Sahlgrenska University Hospital, Gothenburg, Sweden; ^3^ Regional Cancer Center West, Region Västra Götaland, Sahlgrenska Academy, University of Gothenburg, Gothenburg, Sweden; ^4^ Department of Oncology, Institute of Clinical Sciences, Sahlgrenska Academy, University of Gothenburg, Gothenburg, Sweden; ^5^ Department Cardiology, Sahlgrenska University Hospital, Gothenburg, Sweden; ^6^ Department of Molecular and Clinical Medicine, Institute of Medicine, Sahlgrenska Academy, University of Gothenburg, Gothenburg, Sweden; ^7^ Department of Internal Medicine/Respiratory Medicine and Allergology, Sahlgrenska University Hospital, Gothenburg, Sweden

**Keywords:** cancer, immunosuppression, heart transplantation, epidemiology, cohort study

## Abstract

Lung transplantation (LTx) is a well-known treatment for end-stage lung disease. This study aimed to report the incidence of cancer after LTx and long-term outcome among lung transplant recipients with a pretransplant diagnosis of cancer. Patients who underwent LTx between 1990–2016 were included in the study. Detection of cancer was obtained by cross-checking the study population with the Swedish Cancer Registry and the Cause-of-Death registry. A total of 614 patients were followed for a median of 5.1 years. In all, 159 malignancies were diagnosed. The excess risk of cancer or standardized incidence ratio (SIR) following LTx was 5.6-fold compared to the general Swedish population. The most common malignancies were non-melanoma skin cancer (NMSC) (SIR 76.5 (95%CI 61.7–94.8); non-Hodgkin lymphoma (SIR 23.5, 95%CI 14.8–37.2); and lung cancer (SIR 8.89, 95%CI 5.67–13.9). There was no significant difference in overall survival between those with and without a history of cancer before LTx (p = 0.56). In total, 159 malignancies were identified after LTx, which was a 5.6-fold higher relative to the general population. A history of previous cancer yields similar survival in selected recipients, compared to those without cancer prior to LTx.

## Graphical Abstract

**Figure:**
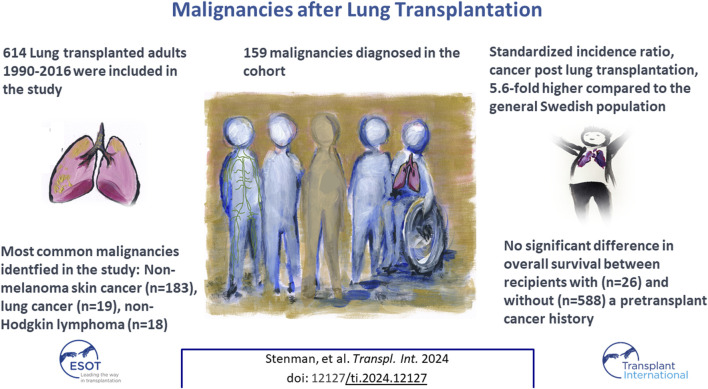

## Introduction

Lung transplantation (LTx) is nowadays an established treatment for patients with end-stage respiratory disease [1]. The immunosuppression is a crucial component to prevent graft rejection after LTx. However, there is a well-known risk of developing cancer in immunosuppressed transplant recipients [2, 3] above all thoracic transplants [4]. After solid organ transplantation, the risk has been reported to increase 2-4-fold compared with the general population [5, 6]. Lung cancer has been reported to be the most common malignancy, excluding non-melanoma skin cancer (NMSC), after LTx [7]. There are several factors associated with the increased risk of developing cancer after LTx, such as: higher age of both donor and recipient; type of immunosuppression; and type of LTx (single or bilateral) [8, 9] but also likely the fact that the 5-year survival rate after LTx has increased from 46% in 1990 to 57% in 2015 [10, 11].

Our study aims to report the incidence and long-term outcome of malignancies after LTx, and to report the outcome in those treated for cancer before LTx.

## Materials and Methods

**FIGURE 1:**
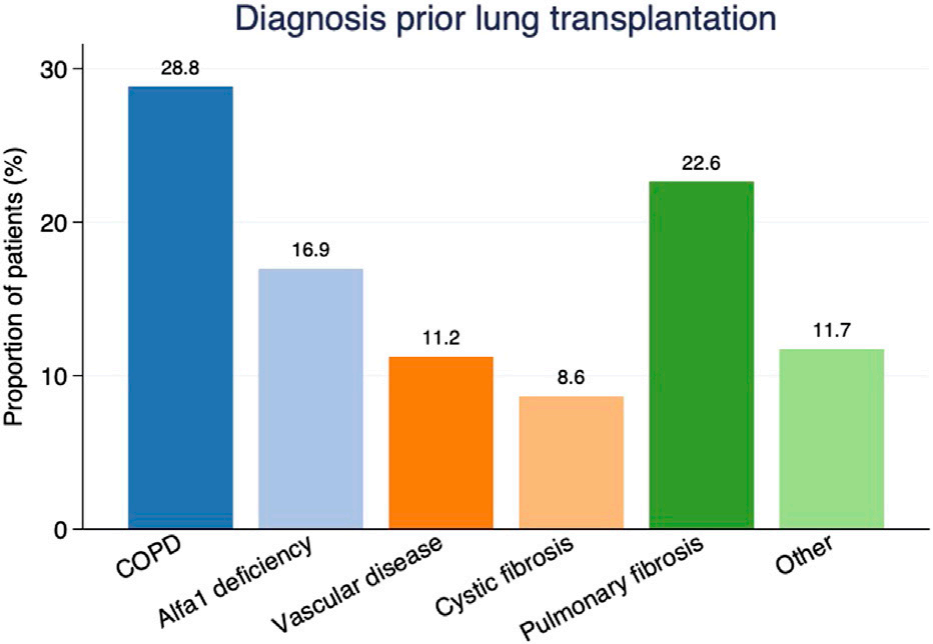
Etiology of lung failure as proportions of patients by diagnosis prior to lung transplantation. COPD, Chronic obstructive pulmonary disease.

From February 1990 to December 2016, 685 LTx were performed in 633 patients at Sahlgrenska University Hospital (SUH) in Gothenburg, Sweden. We used the local patient registry at the Transplant Institute to identify those patients. We excluded those who were not Swedish citizens, and thus did not receive follow-up in Sweden after LTx (n = 19), and those who underwent re-transplantation (n = 52). The remaining 614 patients [266 (43%) men, 348 (57%) women, mean age of 50.0 years] had a median follow-up time of 5.1 years and were included in the study. Baseline characteristics for the cohort are shown in Table 1, also stratified for time era. Chronic obstructive pulmonary disease (177, 29%), pulmonary fibrosis (139, 23%), alfa-1-deficiency (104, 17%) and vascular disease (69, 11%) were the leading indications for lung transplantation in this cohort (Figure 1).

**TABLE 1:** Patient characteristics.

	Total	1985–2000	2001–2010	2011–2016	p-value
	N = 614	N = 162	N = 237	N = 215	
Sex					0.87
Males	266 (43%)	70 (43%)	100 (42%)	96 (45%)	
Females	348 (57%)	92 (57%)	137 (58%)	119 (55%)	
Age at LTx, mean (SD)	50.0 (13.7)	44.5 (12.9)	50.8 (13.2)	53.3 (13.6)	<0.001
Age at LTx, median (IQR)	54.0 (44.0,60.0)	48.5 (36.0–54.0)	55.0 (44.0,60)	56.0 (48.0,63)	<0.001
Age-group at LTx					<0.001
<18	19 (3%)	7 (4%)	7 (3%)	5 (2%)	
18–29	51 (8%)	20 (12%)	16 (7%)	15 (7%)	
30–39	53 (9%)	21 (13%)	21 (9%)	11 (5%)	
40–49	102 (17%)	39 (24%)	37 (16%)	26 (12%)	
50–59	230 (37%)	66 (41%)	94 (40%)	70 (33%)	
≥60	159 (26%)	9 (6%)	62 (26%)	88 (41%)	
Diagnoses					<0.001
COPD	177 (29%)	41 (25%)	78 (33%)	58 (27%)	
α-1-antitrypsin	104 (17%)	42 (26%)	34 (14%)	28 (13%)	
Eisenmenger	26 (4%)	21 (13%)	4 (2%)	1 (0%)	
Pulmonary arterial hypertension	43 (7%)	18 (11%)	14 (6%)	11 (5%)	
Cystic fibrosis	53 9%)	14 (9%)	20 (8%)	19 (9%)	
Pulmonary fibrosis	139 (23%)	16 (10%)	57 (24%)	66 (31%)	
Sarcoidosis	16 (3%)	2 (1%)	8 (3%)	6 (3%)	
Sclerodermia	9 (1%)	0 (0%)	7 (3%)	2 (1%)	
LAM	8 (1%)	0 (0%)	3 (1%)	5 (2%)	
GVHD	7 (1%)	0 (0%)	4 (2%)	3 (1%)	
Bronchiectasis	7 (1%)	0 (0%)	2 (1%)	5 (2%)	
Other	163 (27%)	23 (14%)	63 (27%)	77 (36%)	
Previous malignancy, and type					
Mycosis Fungoides 5	5 (19%)				
Lung	5 (19%)				
Breast	4 (15%)				
Lymphosarcoma/reticulosarcoma	2 (7%)				
Prostate	2 (7%)				
Brain and nervous system	1 (4%)				
Kidney	1 (4%)				
Bladder and urinary organs	1 (4%)				
Female genital organs	1 (4%)				
Lip	1 (4%)				
Lymphoma (reticulosis)	1 (4%)				
Skin (reticulosis)	1 (4%)				
Cervix uteri	1 (4%)				
Total	26				
Smoking					0.15
No, never	201 (33%)	57 (35%)	68 (29%)	76 (35%)	
No, stopped<6 mo. before LTx	5 (1%)	3 (2%)	1 (0%)	1 (0%)	
No, stopped>6 mo. before LTx	391 (64%)	98 (60%)	164 (69%)	129 (60%)	
Missing	17 (3%)	4 (2%)	4 (2%)	9 (4%)	
Transplant type					<0.001
Single transplant	235 (38%)	86 (53%)	126 (53%)	23 (11%)	
Double transplant	379 (62%)	76 (47%)	111 (47%)	192 (89%)	
Donor sex					0.045
Males	296 (48%)	85 (52%)	122 (51%)	89 (41%)	
Females	318 (52%)	77 (48%)	115 (49%)	126 (59%)	
Donor age (median)	46 (30, 57)	34 (21, 47)	46 (33, 57)	53 (40, 63)	<0.001
CMV mismatch					0.11
No (0)	408 (66%)	123 (76%)	153 (65%)	132 (61%)	
Yes (1)	80 (13%)	15 (9%)	36 (15%)	29 (13%)	
Missing	126 (21%)	24 (15%)	48 (20%)	54 (25%)	
BMI	22.1 (19.3, 25.6)	20.5 (18.7, 22.9)	22.4 (19.3, 26.0)	23.2 (19.9, 26.5)	<0.001
Recipient blood group					0.69
0	221 (36%)	59 (36%)	81 (34%)	81 (38%)	
A	294 (48%)	75 (46%)	117 (49%)	102 (47%)	
AB	28 (5%)	11 (7%)	8 (3%)	9 (4%)	
B	71 (12%)	17 (10%)	31 (13%)	23 (11%)	
Follow-up (years) median (IQR)	5.1 (2.2, 9.9)	8.9 (2.0, 17.0)	8.3 (2.8, 11.5)	3.7 (2.0, 5.3)	<0.001

COPD, chronic obstructive pulmonary disease; LAM, lymphangioleiomyomatosis; GVHD, graft versus host disease; CMV, cytomegalo virus.

Digital and scanned medical records from the transplantation unit were reviewed for all the patients. The Swedish Population Registry, which contains complete data about population changes, such as the number of births, deaths, immigration, and emigration, was used. The cancer diagnoses were obtained from the Swedish Cancer Registry (SCR) of the National Board of Health and Welfare, which contains data on all cancers diagnosed among the Swedish population since 1958. Since the start of the registry, it has been mandatory for pathologists and clinicians in Sweden to report cancer diagnosis to the SCR. Therefore, the registry has a very high coverage rate, and all our patients were crosschecked with the SCR [12] and the national Swedish cause-of-death registry to identify all patient’s survival and specifically those with a diagnosis of cancer after LTx. We did not investigate whether the patients have had basal cell carcinoma (BCC), since it is not reported in the SCR, but also since the BCC carcinoma type tends to grow slowly and metastases rarely occur [13].

At our centre, cancer, in general, has been a contraindication for listing for LTx with a few exceptions (n = 26) outlined in Table 1. We have followed International Society for Heart and Lung Transplantation (ISHLT) recommendations, and those with a cancer-free interval of at least 5 years, have been eligible for waitlisting [14]. All of the cancer diagnoses were histopathologically verified. The International Statistical Classification of Diseases (ICD-10) code was used to classify cancers. Our study was conducted in accordance with the Declaration of Helsinki and was approved by the Regional Ethical Review Board at University of Gothenburg (EPN no. 019-09, approval date 22nd Oct 2009, amendments approved 29th Nov 2010, 10th Dec 2012, 17th Dec 2013, 10th May 2017).

The immunosuppressive agents were administered according to a local protocol and has remained relatively unchanged over time. Most of the patients that underwent transplantation at SUH were treated with induction therapy (n = 599) using a t-cell antibody to minimize the risk of early acute rejection [15]. In general, all patients then received a triple combination of a calcineurin inhibitor (tacrolimus or cyclosporine), an antiproliferative agent, (mycophenolate mofetil (MMF) or Azathioprine), and steroids (Prednisone) [16]. The most used calcineurin inhibitor has been cyclosporine, and Azathioprine was replaced as the antiproliferative agent by MMF in 2004-2005.

Data are presented as means and standard deviations, medians and interquartile ranges, or numbers and percentages. Overall survival curves were generated using Kaplan-Meier estimates and comparisons between groups were performed with the log rank test. Relative survival was calculated using the Ederer II method [17]. Mortality data for the general population in Sweden was used to estimate expected survival rates. The mortality data comprised the probability of death for single-year age groups in 1-year calendar period. Cumulative incidence of cancer was analyzed using competing risk methods with death as a competing event [18]. When analyzing cancer incidence for different cancer types, person-years were calculated from date of the transplantation to the first of the following events: diagnosis of the cancer site; death; or end of surveillance period, i.e., 31 December 2018. The standardized incidence ratio (SIR) was defined as the observed number of cancers during the observation time divided by the expected number of cases, using incidence rates from the Swedish population stratified for 5-year age groups (0–4, 5–9, … 80–84, 85-), sex and calendar year. Incidence rates for different cancer sites were used from the NORDCAN project. Furthermore, the coding of cancer followed definitions according to international rules for multiple primary cancers [19]. Univariable and multivariable risk factor analyses were performed by Cox proportional hazards regression model for the development of posttransplant malignancy. The following parameters were tested by univariable analyses: age (per 10 years), sex, body mass index (BMI, <20; 20–30; >30), smoking (never; cessation >6 months before LTx listing; cessation <6 months before LTx listing), diagnostic groups, donor age (per 10 years), donor cytomegalo virus (CMV) +/−, recipient CMV+/−, CMV mismatch and recipient blood group. Significant risk factors identified in the univariate models were tested also in a multivariable model. All statistical tests were two-sided, and a p-value of <0.05 was considered statistically significant. Statistical analyses were carried out with Stata/IC 16.1.

## Results

**FIGURE 2:**
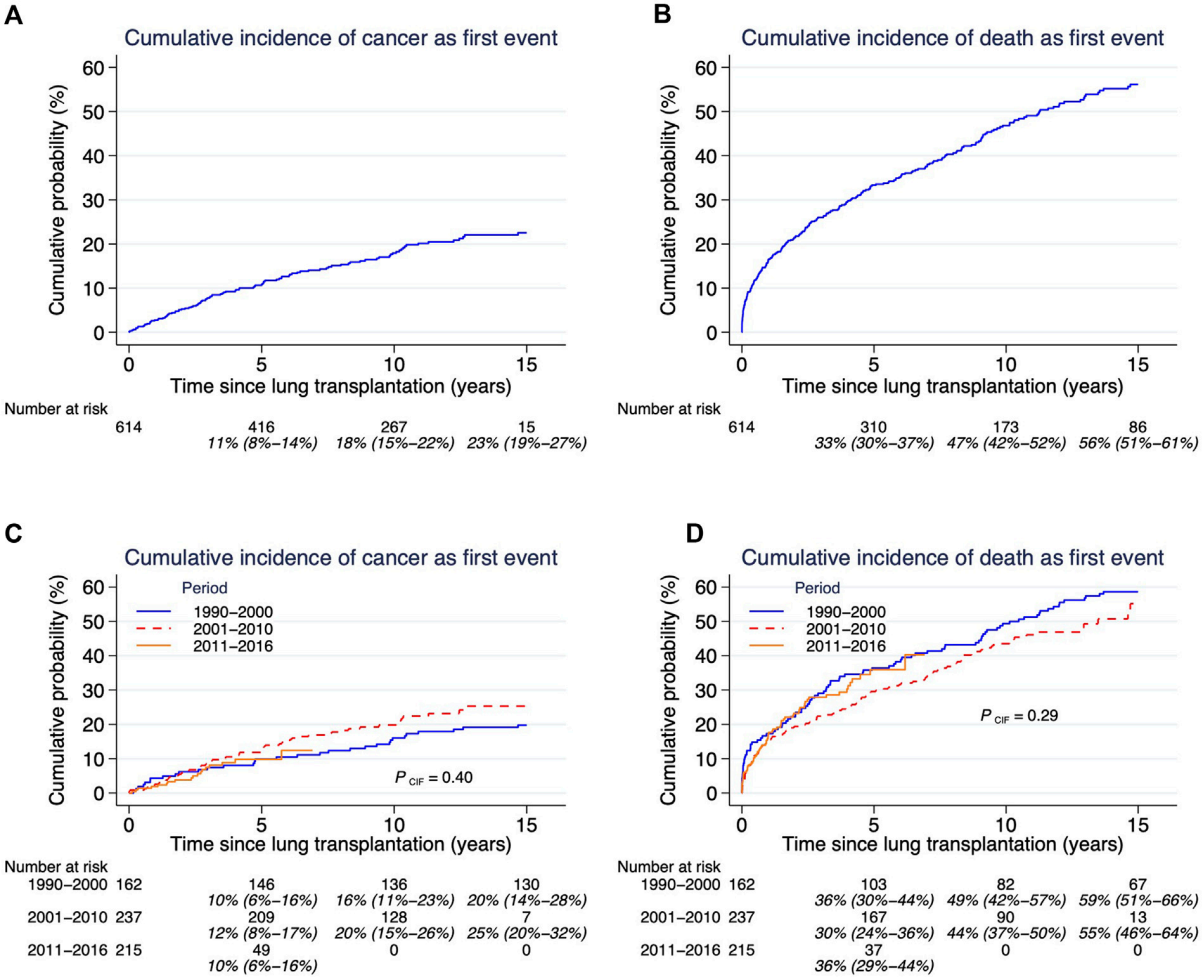
**(A–D)** Cumulative probability of cancer and cumulative incidence of death. Cumulative incidence of competing risks cancer (panel **(A)**] and death [panel **(B)**], and related to time-era after lung transplantation. Both outcomes cancer and death need to be assessed together since they are competing outcomes. Estimates and 95% confidence intervals are shown under the number of patients at risk.

Mortality for the whole patient cohort within 30 days and 1-year post-transplant was 36/614 (6%) and 107/614 (17%), respectively. Overall survival for the whole cohort at 1, 5, and 10 years was: 82% (95%CI 79%–85%); 61% (95%CI 57%–65%); and 43% (95%CI 38%–47%), respectively. Five-year overall survival for those who underwent LTx: was 59% (95%CI: 51%–66%) between 1990 and 2000; 65% (95%CI: 59%–71%) between 2001 and 2010; and 57% (95%CI: 49%–64%) between 2011 and 2016 (Figures 2A–D). There was no significant difference in overall survival over time (p = 0.52). However, risk profile has dramatically changed over time, as illustrated by recipients above 60 years of age were only 6% in the first compared to 41% in the last time-era (p < 0.001) (Table 1).

A total of 159 *de novo* cancers were diagnosed in 111 LTx patients (48 men and 63 women) during follow-up, which corresponds to 18% of the total study population (Table 2). In comparison, 28.6 cancers would have been detected in a cohort from the general population matched by age, sex, and time period. The resulting SIR was 5.56 (95%CI 4.76–6.50) for all cancers after LTx, and 2.76 (95%CI: 2.21–3.46) after excluding NMSC.

**TABLE 2:** Cancer following LTx 1990–2016.

Cancer site (ICD-10)	Sex		Total
	Males	Females	
Extrahepatic bile duct (C24)	0	1	1
Bladder (C67)	2	1	3
Breast (C50)	0	2	2
Colon (C18)	1	1	2
Cervix uteri (C53)	0	1	1
Gallbladder (C23)	1	0	1
Gastric (C16)	1	1	2
Hodgkin lymphoma (C81)	0	2	2
Isthmus uteri (C54)	0	1	1
Lip (C00)	0	2	2
Lung (C34)	6	13	19
Liver cell carcinoma (C22)	1	1	2
Malignant melanoma (C43)	2	3	5
Mouth (C06)	1	0	1
Non-Hodgkin Lymphoma (C83-C85)	4	14	18
Other malignant neoplasms of skin (C44)	39	44	83
Pancreas (C25)	0	1	1
Penis (C60)	1	0	1
Prostate (C61)	8	0	8
Thyroid gland (673)	0	2	2
Vulva (C51)	0	2	2
**Total**	**68**	**92**	**159**

Our study’s cumulative incidence of death was 16.4% at 1 year, 33.3% at 5 years, 46.8% at 10 years and 56.1% at 15 years post-transplantation. Corresponding numbers for the cumulative incidence of cancer were: 2.7% at 1 year; 10.6% at 5 years; 17.9% at 10 years; 22.5% at 15 years; and 23.6% at 20 years (Figures 2A, B). The cumulative incidence of *de novo* malignancy after LTx showed no significant difference between time eras (p = 0.40) (Figure 2C). There was no significant difference in overall mortality (p = 0.29) between the three time periods (Figure 2D).

The type and frequency of solid tumors for the total group, and shown separately for men and women, are listed in Table 2. The most common type of cancer after LTx was non-melanoma skin cancer (NMSC) (52.2% of all cancers), lung cancer (11.9% of all cancers), and non-Hodgkin Lymphoma (11.3% of all cancers). Of those 19 lung cancers that we found after LTx, the majority (n = 13) were developed in the transplanted lung, four in the native lung and two were unknown.

The SIR for cancer types diagnosed in 4 or more individuals are shown in Table 3. The excess risk of all cancers for the total population was 5.6 and similar between men and women (SIR 4.90 and SIR 6.17, respectively).

**TABLE 3:** Observed and expected cancer risks following LTx^a^.

Site (ICDO-10)	Observer number	Expected number	Person years	SIR (95% CI)
All sites				
Total	159	28.6	3903	5.56 (4.7–6.50)
Males	67	13.7	1722	4.90 (3.8–6.23)
Females	92	14.9	2181	6.17 (5.03–7.57)
All sites (exluding NMSC)				
Total	76	27.5	3903	2.76 (2.21–3.46)
Males	28	13.1	1722	2.14 (1.48–3.10)
Females	19	14.4	2181	3.34 (2.51–4.43)
Lung (C33, C34)				
Total	19	2.14	3903	8.89 (5.67–13.9)
Males	6	0.90	1722	6.64 (2.98–14.8)
Females	13	1.23	2181	10.5 (6.12–18.1)
Malignant melanoma (C43)				
Total	5	1.54	3903	3.24 (0.35–7.78)
Males	2	0.71	1722	2.82 (0.70–11.3)
Females	3	0.83	2181	3.60 (1.16–11.1)
Skin, exkl. malignant melanoma (C44)				
Total	83	1.09	3903	76.5 (61.7–94.8)
Males	39	0.58	1722	68.6 (50.1–93.9)
Females	44	0.52	2181	85.1 (63.3–114)
Prostate (C61) Males	8	5.12	1696	1.56 (0.78–3.13)
Non-Hodgkin Lymphoma (C81-C85)				
Total	18	0.77	3903	23.5 (14.8–37.2)
Males	4	0.40	1722	10.0 (3.76–26.7)
Females	5	0.17	2180	38.1 (22.5–64.3)

^a^ Observed and expected number of cancers, person years in follow-up and Standardized Mortality Ratio (SIR) per site after lung transplantation. NMSC, nonmelanoma skin cancer (not including basal cancer).

The overall incidences of cancers in the cohort were higher than expected for: NMSC SIR 76.5 (95%CI 61.7–94.8); non-Hodgkin Lymphoma (SIR 23.5, 95%CI 14.8–37.2); lung cancer (SIR 8.89, 95%CI 5.67–13.9); and malignant melanoma (SIR 3.24, 95%CI 0.35–7.78).

Univariable and multivariable analysis between baseline characteristics and NMSC only is shown in Table 4. Significant risk factors in the multivariable model predicting NMSC only were: age per 10 years [HR 2.23, (95%CI 1.51–3.42), p < 0.001] and recipient blood group A [HR 2.36, (95%CI 1.01–5.53), p < 0.001].

**TABLE 4:** Uni- and multivariable Cox proportional hazard regression for developing NMSC after LTx^a^.

Variable	Number of persons with cancer/N	Cox proportional hazard regression			
		Univariable		Multivariable	
		HR (95% CI)	p	HR (95% CI)	p
Age at LTx, per 10 years	36/614	2.25 (1.51–3.36)	<0.001	2.23 (1.51–3.42)	<0.001
Sex					
Male	16/266	1.0 (ref.)			
Female	20/348	0.98 (0.50–1.88)	0.94		
BMI					
<20	13/196	1.0 (ref.)			
20–30	22/363	1.06 (0.54–2.12)	0.86		
>30	1/50	0.42 (0.05–3.12)	0.40		
Smoking					
No, ended >6 mon before LTx	9/206	1.0 (ref.)			
No, ended <6 mon before LTx	27/391	1.67 (0.78–3.55)	0.50		
Diagnosis group					
COPD	15/177	1.0 (ref.)			
Alfa1 deficiency	9/104	0.96 (0.42–2.19)	0.92		
Vascular disease	0/69	-	-		
Cystic fibrosis	1/53	0.21 (0.03–1.56)	0.13		
Pulmonary fibrosis	9/139	0.96 (0.42–2.19)	0.92		
Other	2/72	0.94 (0.40–2.22)	0.89		
Donor age, per 10 years	36/614	0.34 (0.08–1.50)	0.16		
Recipient blood group					
0	7/221	1.0 (ref.)		1.0 (ref)	
A	22/294	2.46 (1.05–5.76)	0.038	2.36 (1.01–5.53)	0.048
AB	2/28	2.45 (0.51–11.8)	0.18	2.96 (0.61–14.3)	0.18
B	5/71	1.95 (0.62–6.16)	0.72	1.73 (0.55–5.46)	0.35
CMV mismatch					
No	24/408	1.0 (ref.)			
Yes	8/80	1.92 (0.86–4.28)	0.11		

^a^ Time from date of lung transplantation (LTx) to first cancer analyzed. Twenty years follow-up.

LTx, lung transplantation; NMSC, non-melanoma skin cancer; BMI, body mass index; CMV, cytomegalo virus; COPD, chronic obstructive pulmonary disease.

**FIGURE 3:**
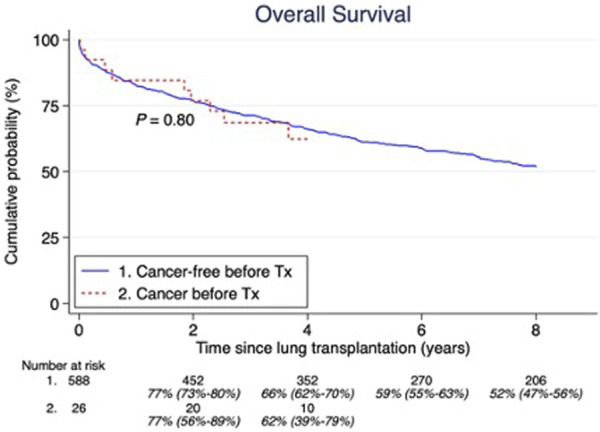
Pretransplant malignancy overall survival compared to those without. Estimates and 95% confidence intervals are shown under the number of patients at risk.

**FIGURE 4:**
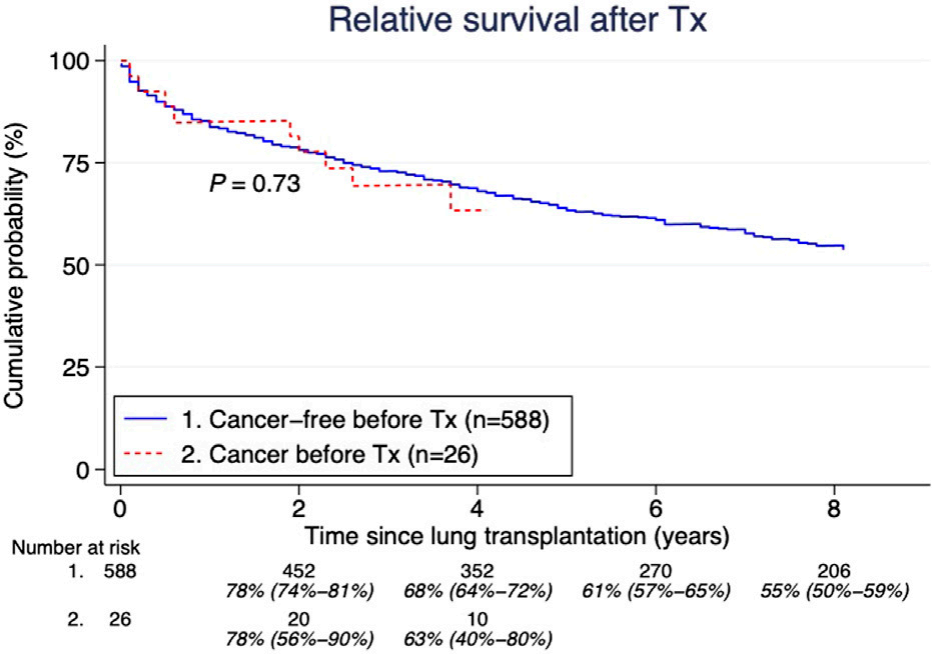
Pretransplant malignancy relative survival compared to those without. Estimates and 95% confidence intervals are shown under the number of patients at risk.

We found 26 (4%) malignancies in 26 patients that had occurred 9.1 years (IQR 2.3–18.3) before LTx. Three of these were actually lung cancers detected at LTx. The median age at their first tumor before LTx was 42 years (IQR 33–53 years), and at LTx the recipient median age was 53 years (IQR 44–59). The most common cancers were: lung, breast, prostate, lymphosarcoma and reticulosarcoma which are shown in Table 1. There was no significant difference in overall survival between those with and without a history of cancer before LTx (p = 0.80) (Figure 3). Furthermore, there was no significant difference (p = 0.73) in post-LTx relative survival between cancer-free patients *versus* those who had experienced cancer before LTx (Figure 4).

We have no data regarding recurrence of cancers in patients who had malignancy before LTx.

## Discussion

This Swedish cohort of lung transplanted patients was studied through national obligatory cancer and cause of death registries, and we observed an overall 5.6-fold excess risk of cancer relative to the general population after a median of 5.1 years of follow-up. The majority of these cancers were NMSC, and when we excluded them from analysis, the risk relative to the general population was reduced to a 2.8-fold excess risk of developing cancer. We also noted, that there was no significantly different survival in patients who had a cancer before LTx, compared to those without a cancer.

Transplanted patients have a higher risk of developing skin cancer. It is also known that those tumors are more aggressive compared to immunocompetent patients [20]. We identified NMSC as the most common cancer, with 83 cancers in a total cohort of 614 patients (13.5%) after LTx. Rashtag et al. identified skin tumors in approximately 28% (47/166 patients) after a median follow-up of 3 years. Berastegui et al. found 39 cases of NMSC in their cohort of 1100 patients (3.5%) after a follow-up of 3 years. However, the data from these studies were not obtained from a national population registry or cancer registry, as in our study, but from individual hospital databases, which might result in missing cancer diagnoses during follow-up. In our study, among NMSC only, age and blood group were identified as independent predictors of developing NMSC after LTx. In concordance Rashtak et al. reported that age per 10 years [HR 1.54 (95%CI 1.14–2.09), p = 0.005] but also male sex [HR 2.79 (95%CI 1.48–5.28), p = 0.002] and a history of NMSC [HR 4.23 (95%CI 1.93–9.26), p =<0.001] were associated with the development of NMSC after lung transplantation [21].

In our study, when we excluded NMSC, the excess risk was 2.8 -fold to develop cancer in relation to the general population. In comparison, a large American study by Magruder et al. observed a 3.26-fold excess risk to develop cancer. In a Spanish study with 1353 patients by Berastegui et al. 125 (9.2%) developed cancer after a mean of 3.7 years, which resulted in a 5-fold higher risk compared to the general population [22]. The cumulative incidence of a *de novo* malignancy between 1 and 5 years after LTx was 7.9%. The most common cancer was NMSC, followed by non-Hodgkin Lymphoma and lung cancer, very similar to our observations. There were no independent predictors of cancer (excluding NMSC) in our study. Predictors of *de novo* malignancies following LTx presented by Magruder et al. were age, male sex and single-lung transplantation. The cumulative incidence of death was in our study 16.4% at 1 year 33.3% at 5 years, 46.8% at 10 years and 56.1% at 15 years post transplantation. Although some report limited survival among lung transplant recipients, Rashtak et al. reported a 53% mortality at 5 years and 86% at 10 years post-LTx. Our survival was better, but clearly lower than for other organ transplants [21]. Corresponding numbers, in a study with over 4000 patients, 5 years and 10 years after liver transplantation was 21% and 33% respectively [23]. A study on kidney transplantation showed a 11% mortality at 5 years and 22% after 10 years. [24]. Obviously, death as an event is a competing outcome to developing cancer, and particularly in LTx with a relatively high cumulative early death rate, the assessment of survival and cancer incidence needs to be analyzed together. Clearly, our study also shows that there was no difference in cumulative incidence of cancer between time eras, and considering that recipients have become older and sicker when accepted for LTx, this might even imply a better screening and detection of cancer in modern era.

After NMSC, non-Hodgkin lymphoma had the highest incidence (SIR 23) of cancer after LTx. A well-known complication after lung transplantation is post-transplant lympho-proliferative disease (PTLD) [25], however this entity does not have its own ICD number, and we account them as non-Hodgkin lymphoma since they are registered in that way by the SCR. In a study by Zaffiri et al. using ISHLT database with more than 19,000 lung transplant procedures registered they were able to identify 454 PTLD, resulting in a cumulative incidence of about 1% during the first year [26]. However, data from this registry was from an international community with a high risk of missing data and not compared to population in general, but our data indicate similar figures, although we cannot discriminate between PTLD and non-Hodgkin lymphomas.

We found a total of 19 lung cancers in 19 patients (3.1%), resulting in a 8.9-fold increased risk after LTx. Corroborating our findings, a French study by Chatron et al. found a total of 19 lung cancers in 463 patients after LTx (4.10%) after a median follow-up time of 21.5 months. [27]. Out of 633 lung transplant patients Pérez-Callejo et al. found that lung cancer was detected in 23 of them (3.63%), with a median follow-time of 3.5 years. [28]. Magruder et al. found the most common cancer after LTx was lung cancer (26.2% of all malignancies, SIR 6.49, 95% CI: 5.04–8.45) after a median follow-up time of 2.97 years. In a large study from the United States, Engels et al. also found an increased risk of developing lung cancer after LTx, resulting in a SIR not very different from ours SIR6.13 (95% CI, 5.18–7.21) [29]. The ISHLT reported that cancer was the 2nd most common cause of death in patients who underwent LTx five to 10 years out from transplant (17.3%) and for patients who were more than 10-year after the procedure (17.9%) [11]. In our cohort, 65% of the patients confirmed a smoking history. Earlier studies have reported that smoking history and older age have been shown to increase the risk of lung cancer after LTx, which are the same risk factors as for the general population. Interestingly, previous smoking was not identified in our study as a predictor of cancer, neither by univariable nor by multivariable testing. Those patients who received a single lung have been reported to be at a higher risk for lung cancer (compared to those who underwent double lung transplantation). The native lung left behind has with inherent risk from the underlying disease, been claimed as a possible explanation [7, 28–32]. Predictors of *de novo* malignancies following LTx presented by Magruder et al. were age, male sex, and single-lung transplantation [7].

## Conclusion

In total 159 malignancies were identified after LTx, which was a 5.6-fold higher relative to the general population. A history of previous cancer yields similar survival in selected recipients, compared to those without cancer prior to LTx.

## Data Availability

The original contributions presented in the study are included in the article/supplementary material, further inquiries can be directed to the corresponding author.

## Abbreviations

BCC, basal cell carcinoma; BMI, body mass index; CI, confidence interval; CMV, cytomegalo virus; COPD, chronic obstructive pulmonary disease; GVHD, graft versus host disease, HR, hazard ratio; ICD, International Statistical Classification of Diseases; ISHLT, International Society for Heart and Lung Transplantation; LAM, lymphangioleiomyomatosis; LTx, lung transplantation; NMSC, non-melanoma skin cancer; MMF, mycophenolate mofetil; PTLD, post-transplant lympho-proliferative disease; SCR, Swedish cancer registry; SIR, standardized incidence ratio; SUH, Sahlgrenska University Hospital.
